# When Checkpoint Inhibition Goes Off Target: A Rare Case of Pembrolizumab-Induced Acalculous Cholecystitis

**DOI:** 10.7759/cureus.109841

**Published:** 2026-05-28

**Authors:** Lan Huynh, Stephanie M Paulson, Walid Shaib

**Affiliations:** 1 Internal Medicine, Wellstar Kennestone Regional Medical Center, Marietta, USA; 2 School of Medicine, Philadelphia College of Osteopathic Medicine - Georgia, Suwanee, USA; 3 Hematology and Oncology, Northwest Georgia Oncology Centers Wellstar, Marietta, USA

**Keywords:** acalculous cholecystitis, adrenal insufficiency, immune checkpoint inhibitor, immune-related adverse event, pembrolizumab

## Abstract

Immune checkpoint inhibitors (ICIs) have revolutionized cancer therapy by improving survival across multiple malignancies by enhancing antitumor immune responses. However, increasing use of ICIs has been accompanied by a growing spectrum of immune-related adverse events (irAEs). Gastrointestinal irAEs most commonly include colitis and hepatitis, whereas biliary complications remain rare and poorly characterized. ICI-associated cholecystitis is an uncommon entity affecting patients treated with ICIs. It often presents as an acalculous disease and occurs months after therapy initiation.

This report describes a case of a 64-year-old woman with lung adenocarcinoma receiving adjuvant pembrolizumab after surgical resection and chemotherapy who developed acute right upper quadrant pain and fatigue during her ninth treatment cycle. Imaging revealed significant gallbladder distension and wall thickening in the absence of gallstones, consistent with acute cholecystitis. Given high operative risk from concurrent ICI-induced adrenal insufficiency and absence of severe inflammatory findings, she was managed conservatively with intravenous fluids, antibiotics, and stress-dose corticosteroids, resulting in clinical improvement. Pembrolizumab was discontinued because of suspected immune-mediated toxicity, and she was discharged with plans for elective cholecystectomy and oncologic surveillance.

This case highlights the importance of maintaining a high index of suspicion for biliary irAEs in patients receiving ICIs who present with abdominal symptoms and underscores the need for multidisciplinary management and further studies to guide optimal treatment strategies.

## Introduction

Immune checkpoint inhibitors (ICIs) targeting cytotoxic T-lymphocyte-associated antigen 4 (CTLA-4) receptor or programmed cell death protein-1 receptor (PD-1) and its ligand (PD-L1) have transformed the management of multiple malignancies, including non-small cell lung cancer (NSCLC), melanoma, breast cancer, and gastrointestinal cancers. Pembrolizumab, a monoclonal antibody, blocks the PD-1 receptor on T lymphocytes, thereby preventing its suppressive effect on the immune response. This mechanism, exploited by cancer cells to evade immune detection, enhances antitumor immunity but also removes regulatory controls, allowing autoreactive T cells to target normal tissues. As a result, immune-related adverse events (irAEs) commonly affect the skin, endocrine organs, gastrointestinal tract, and lungs. Gastrointestinal irAEs typically present as hepatitis, pancreatitis, or enterocolitis, whereas biliary complications, such as acalculus cholecystitis, are rare, with fewer than 30 cases reported in the literature and not specifically addressed in current irAE management guidelines [1-3]. In contrast to calculous cholecystitis, acalculous cholecystitis is much less common, occurs without gallstones, and is typically associated with critical illness and systemic inflammation. This report presents a rare case of acalculous cholecystitis with concurrent adrenal insufficiency in a patient receiving adjuvant pembrolizumab therapy.

## Case presentation

A 64-year-old woman with a history of treated hepatitis C and stage IIIA lung adenocarcinoma, previously managed with surgical resection and four cycles of cisplatin and pemetrexed, was initiated on adjuvant pembrolizumab following tumor profiling that demonstrated PD-L1 expression greater than 50%. She tolerated 200 mg of pembrolizumab every three weeks for approximately five months before presenting on day 22 of her ninth cycle with worsening fatigue, generalized arthralgias, abdominal pain, and poor oral intake for one month. On presentation, she was hypotensive but afebrile. Examination revealed right upper quadrant abdominal tenderness without Murphy's sign.

Initial laboratory evaluation showed a white blood cell count of 8.79 × 10⁹/L, aspartate aminotransferase of 40 U/L, alanine aminotransferase of 15 U/L, alkaline phosphatase of 109 U/L, total bilirubin of 0.7 mg/dL, sodium of 132 mmol/L, and an elevated creatinine level from baseline, consistent with acute kidney insufficiency. Her inflammatory markers were elevated, with a C-reactive protein of 8.0 mg/dL and a sedimentation rate of 37 mm/hr. Due to persistent hypotension in the setting of ICI use, a more thorough endocrine assessment was performed and revealed profound secondary adrenal insufficiency, with a serum cortisol level of 1.0 µg/dL and an adrenocorticotropic hormone level of <5 pg/mL. Thyroid-stimulating hormone was mildly elevated at 4.95 µIU/mL (Table 1).

**Table 1:** Labs on presentation. ACTH: adrenocorticotropic hormone; ALP: alkaline phosphatase; ALT: alanine aminotransferase; AST: aspartate aminotransferase; TSH: Thyroid-stimulating hormone; WBC: white blood cell

Parameter	Result	Reference Range
WBC	8.79 x 10^9^/L	3.50-10.50 x 10^9^/L
ALT	15 U/L	0-33 U/L
AST	40 U/L	0-32 U/L
ALP	109 U/L	35-104 U/L
Total bilirubin	0.7 mg/dL	0-1.2 mg/dL
Sodium	132 mmol/L	136-145 mmol/L
Creatinine	0.95 mg/dL	0.5-0.9 mg/dL
Cortisol, morning	1.0 µg/dL	10-20 µg/dL
ACTH	<5 pg/mL	6-50 pg/mL
TSH	4.95 µIU/mL	0.27-4.2 µIU/mL
C-reactive protein	8.0 mg/dL	<0.5 mg/dL
Sedimentation rate	37 mm/hr	0-29 mm/hr
Blood culture	No growth	-

Computed tomography and ultrasonography demonstrated gallbladder distension and a dilated common bile duct without evidence of inflammatory changes (Figure 1). Magnetic resonance imaging and magnetic resonance cholangiopancreatography showed marked gallbladder distension with mild wall thickening (Figure 2). Hepatobiliary iminodiacetic acid scan showed no gallbladder visualized after four hours, confirming acute cholecystitis. No gallstones were identified on any imaging modality.

**Figure 1:**
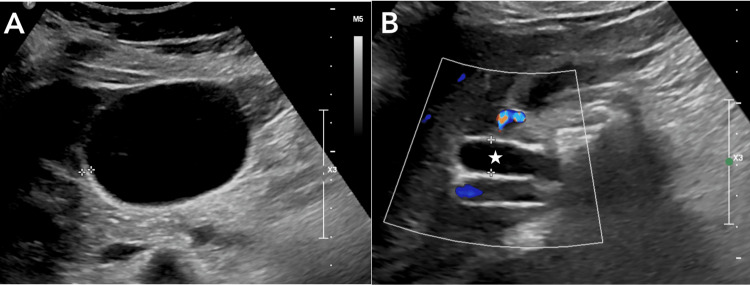
Ultrasonography revealed a moderately distended gallbladder with upper normal limit gallbladder thickness (panel A) and a dilated common bile duct (white star) measuring 11 mm (panel B).

**Figure 2:**
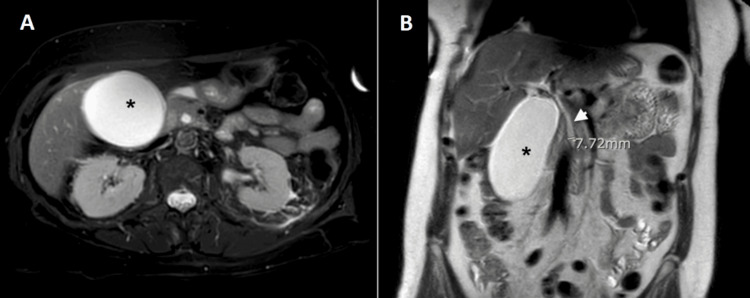
Magnetic resonance imaging revealed marked distension of the gallbladder (black asterisk) with mild wall thickening up to 4 mm (panel A) and a common bile duct (white arrow head) measuring up to 7.7 mm (panel B).

Surgical intervention was deferred due to the high operative risk associated with adrenal insufficiency and the absence of severe inflammatory findings. The patient was managed conservatively with intravenous fluids, empiric antibiotics, stress-dose hydrocortisone, and fludrocortisone, resulting in improvement of pain and resolution of hypotension, acute kidney insufficiency, and hyponatremia. She was discharged on a steroid taper and planned for an elective cholecystectomy. Due to concern for immune-mediated toxicity, pembrolizumab was discontinued, and she proceeded with surveillance.

## Discussion

Pembrolizumab is an FDA-approved ICI that improves overall survival in patients with various cancers and has transformed cancer treatment by enhancing antitumor immunity through PD-1 pathway blockade [2]. However, immune activation is not tumor-specific and may lead to off-target irAEs that affect nearly any organ. Data on ICI-associated cholecystitis remain limited. The strongest evidence linking ICIs to cholecystitis comes from the retrospective series by Abu-Sbeih et al., who identified 25 cases among 4,253 patients treated with ICIs at a tertiary cancer center, corresponding to an incidence of 0.6% [1]. This was significantly higher than the 0.2% incidence of acalculous cholecystitis observed in a matched cohort of cancer patients receiving non-ICI therapy, supporting an association beyond baseline oncologic risk factors [1]. In that study, anti-CTLA-4 monotherapy had a higher incidence than anti-PD-1/PD-L1 monotherapy or combination regimens, mirroring patterns observed with other irAEs such as colitis and hypophysitis [1,2]. Although pembrolizumab-associated cholecystitis appears less common than with CTLA-4 blockade, the growing use of PD-1 inhibitors makes recognition increasingly important.

The clinical presentation of ICI-associated cholecystitis closely resembles conventional acute cholecystitis. In the Abu-Sbeih cohort, abdominal pain was present in 72% of patients, nausea and vomiting in 44%, and fever in 20% [1]. The median time to onset was approximately six months after initiation of immunotherapy, notably later than the 6-12-week timeframe typical for many other irAEs [1]. Our patient developed symptoms after approximately five months of adjuvant pembrolizumab, consistent with this delayed pattern. Importantly, liver enzymes and bilirubin may be minimally elevated or normal, and gallstones are often absent, as in our case, suggesting an inflammatory rather than obstructive process. 

Patients with malignancy often have multiple predisposing factors for gallbladder disease, including advanced age, smoking, obesity, liver metastases, rapid weight loss, and exposure to other potentially biliary-toxic therapies. Nevertheless, the increased incidence in ICI-treated patients, the delayed onset after therapy initiation, and the frequent coexistence of other irAEs support an immune-mediated mechanism. Histopathologic findings from resected gallbladders are generally nonspecific, demonstrating acute and chronic inflammation, mucosal erosion, and pericholecystic fat necrosis rather than a distinctive pattern [1,4]. The concomitant adrenal insufficiency in our patient further supports a shared immunologic pathogenesis.

The clinical course of ICI-associated cholecystitis is variable. Some patients respond to conservative management with intravenous fluids and antibiotics, as observed in this case, while others require invasive interventions. In the cohort described by Abu-Sbeih et al., approximately one-third of patients underwent percutaneous cholecystostomy, and 20%-30% required cholecystectomy due to persistent symptoms or complications such as gallbladder necrosis, perforation, or sepsis [1]. Refractory bile duct obstruction secondary to biliary inflammation is another rare complication, described in a patient on nivolumab who required high-dose steroids, empiric antibiotics, and endoscopic retrograde cholangiopancreatography for drainage and stenting [4]. These findings underscore the unpredictable nature of ICI-associated cholecystitis compared to typical calculous disease, ranging from mild inflammation responsive to supportive care to severe presentations requiring urgent intervention. Our patient improved with intravenous fluids, empiric antibiotics, and endocrine replacement therapy, allowing surgery to be deferred until elective outpatient follow-up.

Most irAEs are effectively managed with corticosteroids or other immunosuppressive agents, such as infliximab, in more severe cases [2]. The role of corticosteroids in the management of ICI-related cholecystitis, however, remains uncertain. In contrast to other irAEs, corticosteroids are not part of the standard treatment for acute cholecystitis, and their direct effect on gallbladder inflammation is unclear. In the largest case series, only 20% of patients received steroids, and steroid exposure was associated with worse overall survival, possibly due to reduced ICI efficacy [1]. However, this finding was likely confounded by poorer baseline performance status and greater comorbidity burden among those selected for steroid therapy [1]. In our case, corticosteroids were clearly indicated for secondary adrenal insufficiency, likely due to immune-mediated hypophysitis. Clinical improvement may therefore have reflected treatment of the concurrent endocrinopathy rather than specific benefit for cholecystitis itself.

Chronic hepatitis C may complicate the interpretation of hepatobiliary abnormalities during ICI therapy. Prior to initiation of pembrolizumab, our patient was diagnosed with genotype 2 hepatitis C, with a viral load of 7.7 million IU/mL. FibroScan evaluation demonstrated no evidence of advanced fibrosis or cirrhosis. She subsequently was started on a 90-day course of sofosbuvir/velpatasvir three weeks prior to the initiation of pembrolizumab. Although ICIs have been associated with immune-mediated hepatitis, available data from a prospective observational study suggest that viral reactivation in patients with treated hepatitis C receiving ICIs for solid tumors is uncommon and occurs only in patients receiving immunosuppressive therapy for management of irAEs [2,5]. In our patient, the absence of significant transaminitis and hyperbilirubinemia during presentation favored an immune-mediated biliary process rather than recurrent viral hepatitis or hepatic decompensation. Additionally, she has sustained virologic response with undetectable viral levels after one month of steroid use for her irAEs.

Another important consideration is whether immunotherapy can be safely resumed after cholecystitis resolves. In the Abu-Sbeih cohort, 40% of patients successfully restarted ICI therapy without recurrence of cholecystitis [1]. More broadly, studies of ICI rechallenge after irAEs in NSCLC patients suggest that approximately half of patients tolerate retreatment without recurrent toxicity, while others experience recurrence of the same irAE or development of a new one [6]. Another study demonstrated that subsequent anti-PD-1 therapy could be safely administered to melanoma patients following severe toxicity due to ipilimumab, a CTLA-4 inhibitor, with relatively low recurrence rates, indicating that toxicity may be treatment-specific rather than class-wide [7]. In reported cases of ICI-related cholecystitis, immunotherapy was often withheld during acute episodes, with permanent discontinuation reserved for severe or recurrent disease [1,4,8,9]. It is unclear whether retreatment at a lower dose is associated with reduced recurrence of toxicity. Given limited experience with cholecystitis, decisions regarding rechallenge should be individualized through multidisciplinary discussion, taking into account the severity of the event, concurrent toxicities, alternative oncologic options, and the anticipated survival benefit of continued immunotherapy. In our patient, pembrolizumab was discontinued because of dual immune toxicities and the relatively favorable oncologic context of adjuvant therapy after definitive treatment.

## Conclusions

This case adds to the limited literature describing pembrolizumab-associated acalculous cholecystitis and highlights several practical lessons. First, clinicians should maintain a high index of suspicion for biliary irAEs in patients receiving ICIs who present with abdominal pain, even when gallstones are absent, and liver tests are normal. Second, because multiple irAEs may occur simultaneously, a comprehensive evaluation for endocrinopathies and other organ toxicities is essential. Finally, management generally parallels standard treatment for acute cholecystitis, with supportive care, antibiotics, and selective procedural intervention, while the role of corticosteroids should be guided primarily by the presence of overlapping immune-mediated conditions. Further prospective studies are needed to better define the pathophysiology, risk factors, and optimal strategies for treatment and immunotherapy rechallenge.

## References

[1] Abu-Sbeih H, Tran CN, Ge PS (2019). Case series of cancer patients who developed cholecystitis related to immune checkpoint inhibitor treatment. J Immunother Cancer.

[2] Postow MA, Sidlow R, Hellmann MD (2018). Immune-related adverse events associated with immune checkpoint blockade. N Engl J Med.

[3] Wang Y, Zhou S, Yang F (2019). Treatment-related adverse events of PD-1 and PD-L1 inhibitors in clinical trials: a systematic review and meta-analysis. JAMA Oncol.

[4] Kashima J, Okuma Y, Shimizuguchi R, Chiba K (2018). Bile duct obstruction in a patient treated with nivolumab as second-line chemotherapy for advanced non-small-cell lung cancer: a case report. Cancer Immunol Immunother.

[5] Yibirin M, Mustafayev K, Hosry J (2023). Immune checkpoint inhibitors suppress hepatitis C virus replication in infected patients with solid tumors. Am J Gastroenterol.

[6] Santini FC, Rizvi H, Wilkins O (2017). Safety of retreatment with immunotherapy after immune-related toxicity in patients with lung cancers treated with anti-PD(L)-1 therapy. J Clin Oncol.

[7] Menzies AM, Johnson DB, Ramanujam S (2017). Anti-PD-1 therapy in patients with advanced melanoma and preexisting autoimmune disorders or major toxicity with ipilimumab. Ann Oncol.

[8] Cho JH, Sun JM, Lee SH, Ahn JS, Park K, Ahn MJ (2018). Late-onset cholecystitis with cholangitis after avelumab treatment in non-small cell lung cancer. J Thorac Oncol.

[9] Ravella B, Pirzada S, Brinkman M, Khaiser A (2023). What made the gallbladder angry? A case of ipilimumab and nivolumab induced acute cholecystitis. Am J Gastroenterol.

